# Graph Theoretical Description of Phase Transitions in Complex Multiscale Phases with Supramolecular Assemblies

**DOI:** 10.1002/advs.202402464

**Published:** 2024-07-01

**Authors:** Ruochen Yang, Kalil Bernardino, Xiongye Xiao, Weverson R. Gomes, Davi A. Mattoso, Nicholas A. Kotov, Paul Bogdan, André F. de Moura

**Affiliations:** ^1^ Ming Hsieh Department of Electrical and Computer Engineering University of Southern California Los Angeles CA 90089 USA; ^2^ Center of Complex Particle Systems (COMPASS) Ann Arbor MI 48109‐2102 USA; ^3^ Department of Chemistry Federal University of São Carlos São Carlos SP 13565‐905 Brazil; ^4^ Department of Chemical Engineering Department of Materials Science and Engineering Biointerfaces Institute University of Michigan Ann Arbor MI 48109‐2102 USA

**Keywords:** complex multiscale phases, graph theory, order parameters, phase transitions, topological metrics

## Abstract

Phase transitions are typically quantified using order parameters, such as crystal lattice distances and radial distribution functions, which can identify subtle changes in crystalline materials or high‐contrast phases with large structural differences. However, the identification of phases with high complexity, multiscale organization and of complex patterns during the structural fluctuations preceding phase transitions, which are essential for understanding the system pathways between phases, is challenging for those traditional analyses. Here, it is shown that for two model systems— thermotropic liquid crystals and a lyotropic water/surfactant mixtures—graph theoretical (GT) descriptors can successfully identify complex phases combining molecular and nanoscale levels of organization that are hard to characterize with traditional methodologies. Furthermore, the GT descriptors also reveal the pathways between the different phases. Specifically, centrality parameters and node‐based fractal dimension quantify the system behavior preceding the transitions, capturing fluctuation‐induced breakup of aggregates and their long‐range cooperative interactions. GT parameterization can be generalized for a wide range of chemical systems and be instrumental for the growth mechanisms of complex nanostructures.

## Graphical abstract

Graphs representing molecules and their long‐range metrics provide an alternative toolbox to study phase transitions in complex multiscale phases with supramolecular assemblies, both for temperature and composition‐induced transitions, as these metrics can identify subtle changes in the structure of complex materials, quantify the amount of each phase, and characterize the structural fluctuations that anticipate phase transitions.

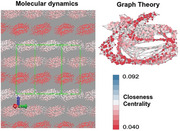

## 1 Introduction

The study of phase transitions is one of the most important subjects in physical sciences. Melting solids and boiling liquids are the most common phase transitions, but more complex structural changes are observed, for instance, in liquid crystals,^[^ ^1^ ^]^ water under extreme pressures,^[^ ^2^ ^]^ hydrocarbons in microemulsions^[^ ^3^ ^]^ and liquid‐liquid transitions in ionic liquids.^[^ ^4^ ^]^ Phase diagrams may be mapped by X‐ray diffraction,^[^ ^5^, ^6^ ^]^ light scattering,^[^ ^7^ ^]^ Raman spectroscopy^[^ ^8^ ^]^ and nuclear magnetic resonance.^[^ ^9^ ^]^ Concurrently, Monte Carlo (MC)^[^ ^10^, ^11^ ^]^ and molecular dynamics (MD) simulations^[^ ^12^, ^13^ ^]^ have become remarkably accurate and can predict structural differences between solids and liquids for specific phase transitions.^[^ ^14^, ^15^, ^16^, ^17^ ^]^

Various structural, thermodynamic and topological parameters are employed to identify individual phases, e.g., degree of crystalline order, organizational symmetry, or interatomic distances, which are described in terms of crystal unit cells' dimensions and symmetries, radial distribution functions (RDF), and orientational order parameters defined by Legendre polynomials or spherical harmonics. However, these phases are complex, i.e., combining both order and disorder. Additionally, complex phases can reveal organization and nonrandom (correlated) disorder at different scales. Previously used order parameters well suited for crystalline phases, become less descriptive for phases with greater degree of disorder or hierarchical organization over multiple length scales. The latter can be exemplified by the complex microparticles formed by self‐assembly of chiral gold sulfide into 2D nanosheets via supramolecular interactions; they have nearly identical lattices at atomic scale but markedly different packing of the nanosheets in nano‐ and microscale.^[^ ^18^ ^]^ Similar problems also emerge for complex phases of high molecular weight amphiphilic molecules, polymers, ionic liquids, and nanoparticles, which display some of the richest and most complex known phases,^[^ ^19^, ^20^, ^21^, ^22^, ^23^, ^24^, ^25^, ^26^, ^27^ ^]^ dealing with complex, i.e., combining simultaneously order and disorder, phases.^[^ ^28^ ^]^ Furthermore, even seemingly simple perfectly crystalline or perfectly disordered phases may have structures that deviate from classical descriptions and require multiple organization levels to be considered.^[^ ^29^, ^30^, ^31^ ^]^

The transient states between these phases are also essential. From the fundamental standpoint, they display complex organizational patterns that are difficult to quantify. From the practical standpoint, better understanding of the transient states helps identifying the pathway to complex phases with unique mechanical, electrical, optical, biological and other properties. The key problem here is that the transient states, again, involve some degree of order and disorder,^[^ ^32^ ^]^ which is difficult to describe using the traditional order parameters, lattice models,^[^ ^33^, ^34^, ^35^ ^]^ the Maier‐Saupe theory,^[^ ^9^ ^]^ or knot‐based topological parameters.^[^ ^17^ ^]^

Here, we evaluate how descriptors of phase connectivity based on graph theory (GT) can be utilized for addressing these problems. GT offers a mathematical toolbox to quantify structural changes of complex phases over both long and short length scales, describing the entire network of interacting molecules,^[^ ^16^, ^36^, ^37^, ^38^, ^39^, ^40^, ^41^ ^]^ adequately accounting for polydispersity of particles or molecules^[^ ^42^ ^]^ and capturing the simultaneous presence of order and disorder in transient states close to the transition region.^[^ ^43^ ^]^ As model systems, we have chosen the ionic thermotropic liquid crystal 1‐hexadecyl‐3‐methylimidazolium tetrafluoroborate (C16MIMBF4) and the binary solutions of *n*‐octyltrimethylammonium bromide (OTAB) in water, in the composition range forming spheroidal micelles (isotropic phase) and two lyotropic mesophases formed by cylinders (hexagonal phase), and lamellae (lamellar phase), mapping the phase diagram with MD simulations in all cases. GT parameters, such as centrality measures and node‐based fractal dimension (NFD),^[^ ^44^, ^45^ ^]^ were compared to RDF and Legendre polynomials of the second degree (*P*_2_), commonly used for phase description and localization of phase transition.

## 2 Results and Discussion

### 2.1 Temperature‐Induced Phase Transitions

**Figure 1:**
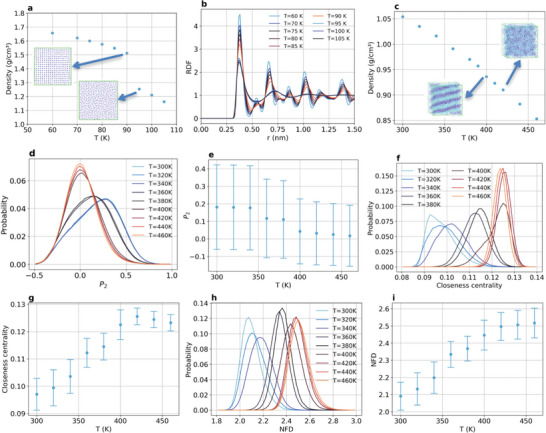
Transitions between complex phases with multiscale organization induced by temperature. a) Average density and b) radial distribution function (RDF) of argon around the solid‐liquid phase transition (insets in (a) show the final structures obtained before and after the phase transition). The average values were calculated over 100 ns using 10⁵ configurations (error bars have been omitted because the largest standard deviation calculated for the density was only 0.004 g cm^−^^3^). c) Average density of C16MIMBF4 around the liquid crystal‐isotropic liquid phase transition (insets show the final structures obtained before and after the phase transition). The average values were calculated over 100 ns using 10⁴ configurations (error bars have been omitted because the largest standard deviation calculated for the density was only 0.0012 g cm^−^^3^). d) Probability distributions and e) average values of *P*_2_ order parameter for the vectors along the aliphatic chains of the C16MIM cation. f) Probability distributions and g) average values of closeness centrality considering each aliphatic chains of the C16MIM cation as a node in the graph. h) Probability distributions and i) average values of node‐based fractal dimension (NFD) considering each aliphatic chains of the C16MIM cation as a node in the graph.

The identification of transitions between complex phases with multiscale organization depends on the measurement and/or calculation of properties whose values differ appreciably in the two phases involved in the transition. These properties will be referred to as metrics and will be considered as order parameters herein since the changes in values of each metric as the systems undergo phase transitions is related to changes in atomic/molecular ordering. For instance, one of the simplest phase transitions is the melting of solid argon, which is accompanied by a large change in density (**Figure** **1**a). The MD simulations overestimate the melting temperature and underestimate the densities as compared to experimental results, but they capture the physics of the process, which can be further detailed by the RDF changes as the temperature increases, leading to the decrease in correlation peaks’ heights and even the suppression of secondary peaks typical of the face‐centered cubic (FCC) lattice as the system crosses the transition temperature (Figure 1b). Before the melting, the *g*(*r*) peaks get broader with the temperature as the amplitude of the atomic vibrations in the crystal increases and some instantaneous distortions can be noticed in the local structure around any selected atom, but with the melting both the local close packing and the long‐range structure characteristic of the crystal is lost (Figure S1a, Supporting Information). Since RDFs are local densities *ρ*(*r*) normalized with respect to the average densities *ρ*_0_ depicted in Figure 1a, both metrics can properly locate phase transitions characterized by changes in atomic/molecular packing, but they are not useful when packing does not change appreciably, as in the case of liquid crystal‐isotropic liquid transitions (Figure 1c), since liquid crystals lack positional order just like the isotropic liquids, but retain some degree of orientational order, leading to similar densities for both phases, even though the structure of the phases differ markedly (inset in Figure 1c). The metric most often used for experimentally assessing liquid crystals phase transitions is the Legendre polynomials of the second degree (*P*_2_), which captures the relative orientation between representative vectors of the structural units comprising the system. Taking the tail of the aliphatic chains of the 1‐hexadecyl‐3methylimidazolium (C16MIM) cation as the reference vector for each molecule and computing the average cosine between neighboring molecules vectors yields broad distributions of *P*_2_ values which change with temperature (Figure 1d). The spread of each distribution covers nearly all the available values for *P*_2_ from −0.5 to 1.0, leading to standard deviation values much larger than the changes observed for the mean *P*_2_ at each temperature (Figure 1e). Topological metrics may be short‐ranged, like degree centrality and clustering coefficients,^[^ ^48^ ^]^ or long‐ranged, like closeness centrality and node‐based fractal dimension (NFD).^[^ ^43^, ^44^ ^]^ The former resemble local densities and, as such, they can only capture large structural changes as those observed for the FCC‐liquid transition of argon (Figure S1a, Supporting Information). On the other hand, the later can capture subtle structural changes taking place for the liquid crystal‐isotropic liquid transition, since both closeness centrality and NFD distributions shift to higher values as the temperature increases (Figure 1f,h). The relative broadness of these distributions is smaller than those observed for *P*_2_ (Figure 1d), leading to smaller standard deviations values (Figure 1g,i). It is important to acknowledge that *P*_2_ (Figure 1e), closeness centrality (Figure 1g) and NFD (Figure 1i) all indicate that the liquid crystal‐isotropic liquid transition is not sharp for C16MIMBF4, being characterized by either a smooth decrease (*P*_2_) or a smooth increase (closeness centrality and NFD) of the average value of each metric toward plateaus when the transition is complete. This is not a limitation of the metrics, but rather a characteristic of this system. Regarding the simple solid‐liquid transition in argon, both short and long‐ranged GT metrics can easily capture the phase transition (Figure S1b–d, Supporting Information) and, as in the case of the liquid crystal, not only the average values of closeness centrality and NFD changes with the transition, but the broadness of their distributions change as well.

**Figure 2:**
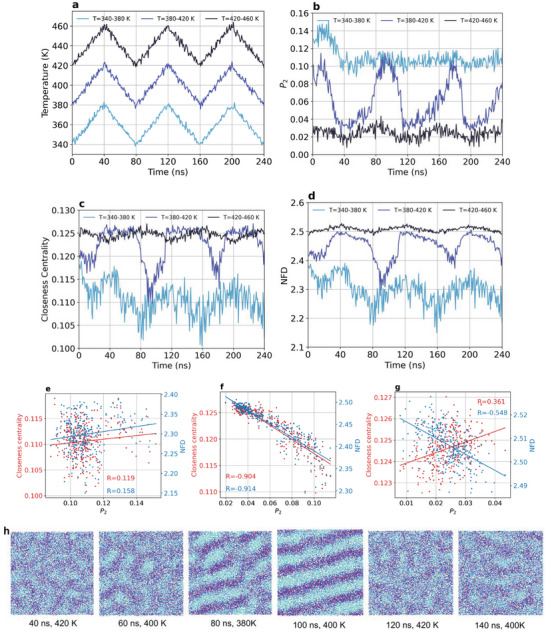
Cyclic phase transitions induced by temperature. a) Cyclic heating and cooling of C16MIMBF4 at temperature ranges below (*T* = 340–380 K), around (*T* = 380–420 K) and above (*T* = 420–460 K) the liquid crystal‐isotropic liquid phase transition. Average values of b) *P*_2_ order parameter, c) closeness centrality and d) node‐based fractal dimension for the aliphatic chains of the C16MIM cation. Correlation between GT metrics and *P*_2_ order parameter for the temperature scan e) below, f) around, and g) above the transition region. h) Selected snapshots from the heating and cooling cycles between 380 and 420 K showing the structural hysteresis in the phase transition. The respective times and temperatures are given below the structures where aliphatic groups are displayed in cyan, the charged portion of the cation in dark blue and anions in red.

Both topological and orientational metrics are responsive to temperature changes during cyclic heating and cooling of the mesogenic substance C16MIMBF4 (**Figure** **2**a–d). The *P*_2_ order parameter had only low amplitude responses when the temperature changes below or above the transition region, while larger amplitude responses occur if the heating and cooling cycles cross the transition region around 400 K (Figure 2b). Closeness centrality and NFD also had only small changes for the isotropic liquid above the transition region, but presented large changes for both the liquid crystal and for the transition region (Figure 2c,d). In the transition temperature range, *P*_2_ relaxes faster than GT metrics, which indicates that, in a non‐equilibrium situation such as the fast heating/cooling performed here, short‐range orientation tends to relax faster than the long‐range connectivity captured by both closeness centrality and NFD, rendering the location of the phase transitions at different times/temperatures dependent on the metric used. However, this inconsistency cannot be used to favor one metric or the other as the best one to capture the phase transition, but they give complementary information regarding the system relaxation in a non‐equilibrium situation in which the location of a transition will always be somewhat arbitrary since the structure is evolving in time. If the temperature is changed stepwise and the system can achieve equilibrium at each temperature, as in Figure 1d–f, both *P*_2_ and long‐range GT metrics capture the same temperature for the phase transition. The weak correlations between GT metrics and *P*_2_ in the single phase regions (Figure 2e,g) and the large correlations observed as the system switches back and forth between the liquid crystal and the isotropic phase (Figure 2f) demonstrate that topological metrics, although lacking information about the relative orientation between neighboring molecules, can capture the phase transitions just like *P*_2_ orientational order parameter, indicating that liquid crystal‐isotropic liquid transitions are not characterized only by the loss of orientational order, but also by changes in long‐range order beyond first shell neighbors.

### 2.2 Composition‐Induced Phase Transitions

Besides the temperature, phase transitions can also be observed due to changes in the composition in aqueous solutions of amphiphilic species and those can also be characterized by GT. OTAB and water molecules were described by coarse‐grained models comprising one charged hydrophilic site (head) and two hydrophobic sites (tail) for the surfactant and a single site representing solvent molecules. Computational models, referred to as real solutions, were used because they effectively describe the anisotropy of OTAB interactions, which is essential for the formation of surfactant aggregates^[^ ^46^ ^]^ and other supramolecular assemblies. We also carried out simulations with surfactant molecules interacting isotropically with each other and with water, setting the interaction parameters for OTAB sites identical to those of water particles. Such models, referred to as ideal solutions, are unphysical but provide a convenient baseline for understanding the relations of GT parameters to intermolecular interactions. For example, the uniform distribution of particles and their GT descriptors in space indicates that no self‐assembly or phase transition was observed (Figures S2 and S3, Supporting Information), as expected for systems with isotropic interactions.

**Figure 3:**
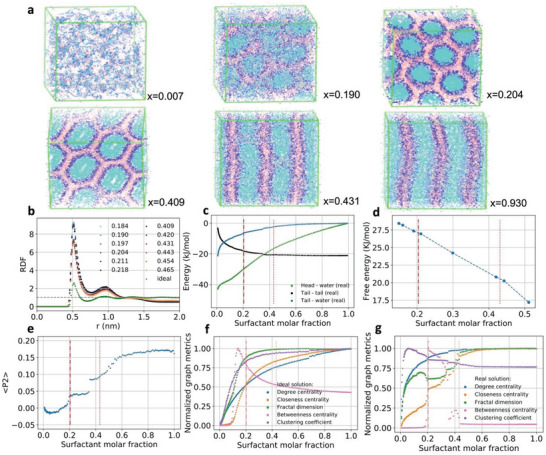
Phase transitions induced by changes in composition. a) MD simulations of OTAB/water system showing different phases observed at specific surfactant molar fractions. Each representation corresponds to the superposition of 10 structures taken from the end of the respective simulations (cyan—surfactant tail; blue—surfactant head; pink—counter‐ions; water molecules have been omitted for clarity). b) Radial distribution function (RDF) for terminal tail beads for ideal solutions (green curve) and around phase transitions for real solutions at different surfactant molar fractions. c) Average value for selected interaction energy components. d) Free energy changes between real and ideal solutions for selected compositions in the region of the phase diagram with the largest structural changes between different phases. e) Average *P*_2_ order parameter calculated for the relative orientation of the OTA^+^ tails within 0.7 nm of each other. f) Average degree centrality, closeness centrality, betweenness centrality, clustering coefficient, and fractal dimension (NFD) for the ideal solutions (metrics are normalized between 0 and 1 for better visualization). g) Normalized graph metrics for real solutions. The transitions from micellar phase to hexagonal phase at *x* = 0.204 and from hexagonal to lamellar phase at *x* = 0.431 are indicated by vertical red lines in (c–g).

The phase transition was induced by the progressive removal of water encompassing a total of 170 different compositions, gradually increasing OTAB concentrations from surfactant mole fraction *x* = 0.007 to *x* = 1.000 (Table S1, Supporting Information). Two distinct phase transitions were identified in MD models of real solutions: one from isotropic micellar suspension to hexagonal phase of cylindrical micelles at *x* = 0.204 and another one from hexagonal phase to lamellar phase at *x* = 0.431 (**Figure** **3**a). Contrary to the classical picture of phase transitions, the potential energy of tail‐tail, tail‐water, and head‐water interactions had no discontinuity or inflection close to these phase transitions (Figure 3c). In fact, it is known from differential scanning calorimetry (DSC) experiments that the enthalpy changes involved in those transitions are very small and often cannot be captured.^[^ ^47^ ^]^ The free energy difference between the real and ideal solution can be computed by alchemical simulations in which the interaction parameters are changed at different compositions (Figure 3d). Since this method is computationally very expensive, it was performed only for a few compositions around the transitions and a small inflection change is noticed after the transition to the lamellar phase, but no significant differences were noticed due to the transition between isotropic and hexagonal phase, which further emphasize the difficulties of using thermodynamic properties as metrics to describe the phase transitions for systems like this. RDFs computed for the terminal beads in the tails displayed distinct first peaks followed by the oscillatory decay at longer distances, typical of the partial segregation of surfactants into aggregates. However, RDFs changed continuously with the surfactant concentration in the vicinity of the phase transitions (Figure 3b). The RDFs for the ideal solutions displayed no sign of aggregation and the profiles were not dependent on the surfactant concentration, consistent with a random distribution of molecules. The order parameter described by the second rank Legendre coefficient P_2_ exhibited plateaus for each phase and, as such, might in principle identify successfully hexagonal and lamellar phases in OTAB/water system (Figure 3e). However, three observations demonstrate that P_2_ may not be the best choice to map these phase transitions. 1) The point of highest gradient in *P*_2_ at *x* = 0.357 is supposed to indicate the phase transition, but this concentration is far from the two observed transitions. 2) Both hexagonal and lamellar phases should have order parameters larger than the nematic phase, for which Onsager and Maier‐Saupe theories predict *P*_2_ equal to 0.84 and 0.44, respectively. While Onsager theory overestimates the difference between isotropic and nematic phases, the Maier‐Saupe theory is not likely to do so. 3) Since *P*_2_ is more sensitive to changes in the shape of the micelles from circular to cylindrical taking place for *x* between 0.2 and 0.4 (Figure 3a) than to the surfactant packing within micelles.

GT parameters can describe phases and identify phase transitions more accurately than traditional order parameters by taking into account the diversity of aggregate shapes, the dynamics of the molecular segments and their collective interactions over time and space. Importantly, mathematical apparatus of GT affords fast calculations of parameters both on microscopic (local) and macroscopic (global) scales. Considering that surfactant aggregation results in the contact between hydrophobic tails, each surfactant can be considered as a node and two nodes are connected to each other if at least one pair of tail sites between neighboring molecules are closer than a cutoff distance, which was defined as 0.7 nm, corresponding to the first minimum of the RDF for tail sites (Figure 3b). The same criteria was used in GT representations of proteins and their supramolecular complexes with nanoparticles.^[^ ^48^ ^]^

MD trajectories were analyzed using GT for all concentrations as the aggregates changed from spherical micelles to infinite hexagonally packed cylinders, and then to stacked lamellae (Figures S2 and S3, Supporting Information). Degree centrality, closeness centrality, betweenness centrality, clustering coefficient, and NFD^[^ ^43^, ^44^, ^49^ ^]^ were calculated for each node at each frame of each MD simulation for both ideal and real solutions (Figure 3f,g). The phase transition from the micellar to the hexagonal phase at *x* = 0.204 is associated with sharp changes in closeness centrality, betweenness centrality, and NFD, reaching plateaus after the phase transition between the hexagonal and the lamellar phases at *x* = 0.431 (Figure 3g). These findings clearly demonstrate the high sensitivity of these GT descriptors to the different aggregation patterns. We also note that GT metrics that characterize only the immediate surroundings of nodes, as exemplified by degree centrality and clustering coefficient, display poor sensitivity to observed phase transitions. As an additional benchmark, the same GT metrics displayed no singularities for ideal solutions since no phase transition took place (Figures S2 and S3, Supporting Information).

**Figure 4:**
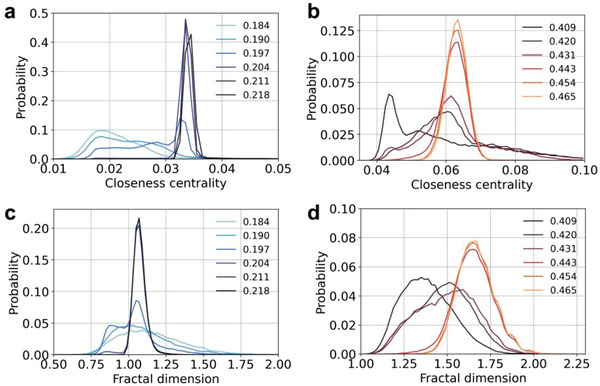
Graph theory metrics. Probability distribution of a,b) closeness centrality and c,d) node‐based fractal dimension (NFD) for different OTAB molar fractions close to a,c) the micellar‐hexagonal and b,d) hexagonal‐lamellar phase transitions. Phase transitions occur at *x* = 0.204 and *x* = 0.431.

The closeness centrality and NFD change from relatively broad distributions to sharp peaks centered at 0.035 and 1.1, respectively, as small micelles rearrange into cylinders (*x* = 0.204 in **Figure** **4**). Further increase in the surfactant concentration broadened all the distributions, which displayed longer tails toward larger values as the cross‐sectional shape of the cylinders of hexagonal phase changed from circular to hexagonal (*x* = 0.204 and *x* = 0.409 in Figure 3a). This shape change led to less ideal graphs, as captured by the closeness centrality and NFD analyses, resulting in the non‐monotonic change of their average values (Figure 3g), followed by the shift of the distributions from left to right as the system undergoes the transition from the hexagonal to lamellar phase (*x* = 0.431 in Figure 4). These distributions are time averages computed over 30 ns for each composition, but the instantaneous values may vary considerably as the systems approach the transition region, while after transition the distributions are stable (Figure S5, Supporting Information), indicating that structural fluctuations take place at the transition regions.

### 2.3 Structural Fluctuations Preceding Phase Transitions

**Figure 5:**
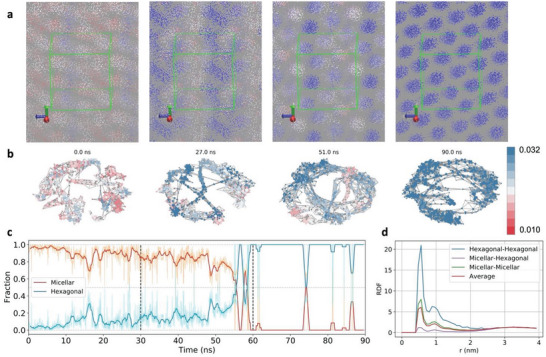
Transition between micellar and hexagonal phases. a) Simulation snapshots taken in the region of the transition from micellar to hexagonal phase showing the surfactant tails with colors based on the instantaneous values of closeness centrality. Periodic replicas were included for better visualization, with the green lines marking the edges of the actual simulation box. b) Graph representations of the phases in (a) with the same color scale. c) Time‐dependent composition of the system given as molar fraction of surfactant for each phase close to the transition; simulations with surfactant molar fraction ranging from 0.190 to 0.204. Red and blue curves are the 25 points running average of the instantaneous data. The vertical dashed line indicates the times at which water particles removal was performed, increasing the surfactant concentration. d) RDFs calculated for the different phases observed using closeness centrality values as described in the text, for *x* = 0.197.

**Figure 6:**
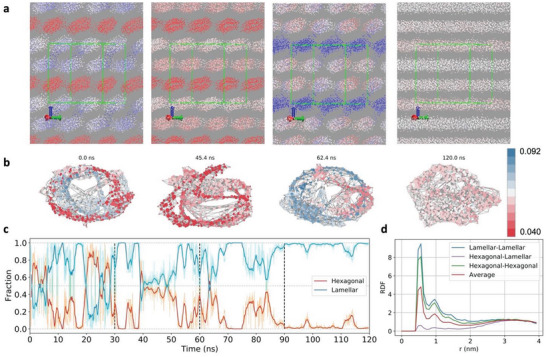
Transition between hexagonal and lamellar phases. a) Simulation snapshots taken in the region of the transition from hexagonal to lamellar phase showing the surfactant tails with colors based on the instantaneous closeness centrality value. Periodic replicas were included for better visualization, with the green lines marking the edges of the actual simulation box. b) Graphs of the same phases as in (a) with the same color scale. c) Time‐dependent composition of the system given as molar fraction of surfactant for each phase close to the transition; simulations with molar fraction ranging from 0.409 to 0.443. Red and blue curves are the 25‐points running average of the instantaneous data. The vertical dashed line indicates the times at which water particles removal was performed, increasing the surfactant concentration. d) RDFs calculated for the different phases observed using closeness centrality values at *x* = 0.420.

Closeness centrality and NFD for compositions close to OTAB phase transitions displayed two populations (Figure 4), consistent with the coexistence of two phases (**Figure** **5**a), which was not static but changed over time (Figure S5, Supporting Information). From the physical standpoint, micelles begin to align to form cylinders. However, strong structural fluctuations accompany this process when the cylindrical clusters break apart and form micelles again, which is reflected by the fluctuations in the closeness centrality. The corresponding graphs and red‐white‐blue colored GT maps show the coexisting structures with different values of closeness centrality (Figure 5a,b). When the cylinders are forming, the graph of molecules becomes more homogeneous, as the nodes transform into uniform blue color with similar closeness centrality (Figure 5a,b). The graph representations exhibit ring‐like structures due to the periodic boundary conditions, which connect different cylinders inside the simulation box into a single periodic superstructure. Similar trends were also observed for the transition between hexagonal and lamellar phases (**Figure** **6**). We note that the molecular clusters in MD snapshots do not necessarily correspond to the node clusters in the graph visualizations. Also note that the graph representations of the ideal solutions displayed only random profiles (Figure S3, Supporting Information), consistent with the absence of segregation.

The probability distributions of closeness centrality depend on the amount of surfactant in each phase at each frame of the MD simulation (Figure 4a and Figure S5, Supporting Information). The closeness centrality values below 0.03 are characteristic of the micellar phase, those between 0.03 and 0.055 correspond to the hexagonal phase, and those above 0.055 correspond to the lamellar phase. The fraction of surfactant of each phase was calculated as a function of time for the regions of the phase diagram close to the observed transitions (Figures 5c and 6c). Both transitions are accompanied by fluctuations in the fractions of each coexisting phase with periods of 3–5 ns, evolving towards a single dominant phase, consistent with the observed unimodal distributions after phase transition (Figure 4). The sharp peaks observed after 60 ns for the transition from micellar to hexagonal phase are due to local breakups of the self‐assembled nanoscale cylinders, yielding smaller closeness centrality values transiently. Molecules belonging to each coexisting phase are clustered together, which was confirmed by the RDFs between the terminal tail site of the surfactant molecules (Figures 5d and 6d). Also, the changes in RDF peaks’ positions are small and not phase‐specific.

**Figure 7:**
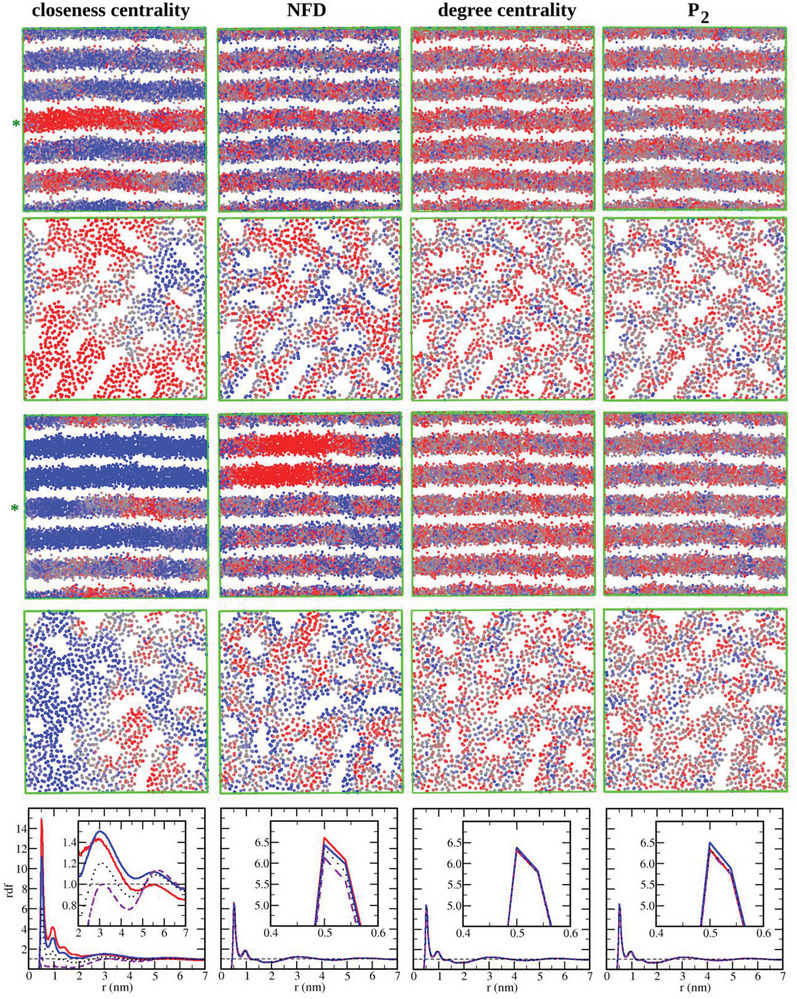
Order parameters. Two selected structures for the larger model system with the OTAB molar fraction *x* = 0.443 showing only the surfactant tail sites colored using (from left to right) closeness centrality, NFD, degree centrality and *P*_2_ values, with red and blue colors representing low and high values of the metrics. Rows 1 and 3 lateral views of the whole system at two different time steps. Rows 2 and 4—the cross‐sectional views of the lamellae marked by the green stars on the left of rows 1 and 3. Molecules close to the cavities in the lamellae tend to present smaller values for both *P*_2_ and degree centrality as compared to molecules farther from the holes, which display a denser first shell of other surfactant molecules. Bottom panel—RDF curves for the terminal tail site of surfactant molecules with red curves including the 25% molecules with lowest values for the metric, blue curves including only the 25% with highest values, purple dashed curves give the distribution of the 25% higher in relation to the 25% lower and the dotted black curves include every surfactant molecule.

The long‐range GT metrics surveying the entire graph should be able to distinguish different structural patterns inside a given phase even for concentrations far from the phase transitions. Indeed, surfactant molecules with closeness centrality specific to the lamellar phase form distinctly segregated regions, with surfactant ions in regions with more holes or larger holes inside the lamellae presenting smaller values of closeness centrality (red color in **Figure** **7**). The NFD had a limited capacity of detecting different regions and degree centrality and *P*_2_ values were nearly homogeneous, as confirmed by RDFs (Figure 7 bottom). Similar results were observed for the hexagonal phase (Figure S6, Supporting Information).

One of the reasons for the increased sensitivity of closeness centrality to subtle structural variations is that this GT descriptor is affected by the transient contact between the structural units, i.e., lamellae, which leads to larger values (Figure 7). The closeness centrality for the hexagonal phase distinctly decreases or increases whenever a cylinder undergoes a partial breakup, which is very convenient for tracing the phase transition pathways (Figure S6, Supporting Information). In comparison, NFD captures the long‐range structural fluctuations of the hexagonal phase but not those in the lamellar phase. The degree centrality and P_2_ predominantly consider the first coordination shell of the molecules, and thus they are only sensitive to short range structural changes.

### 2.4 Node‐Based Multi‐Fractal Analysis (NMFA)

Similarly to the application of Boltzmann statistics to atoms, the time‐variable structure of the molecular assemblies undergoing a phase transition into a nanostructured phase may be considered as a superposition of random graphs with a spectrum of different parameters determining the probability of a specific snapshot. The statistical nature of molecular architectures embedded in the GT representations may be accounted for by node‐based multi‐fractal analysis (NMFA),^[^ ^44^ ^]^ which affords us to simultaneously embed scale information in the graphs due to the box‐growing methodology^[^ ^44^ ^]^ in fractal analysis. In terms of the Lipschitz‐Hölder exponent *α* and multifractal spectrum *f(α)*, the former reflects differences in organizational patterns of nodes emerging at different scales (i.e., box sizes) while the latter reflects the variability of fractality needed to accurately describe the system (i.e., spread of fractal dimensions).

**Figure 8:**
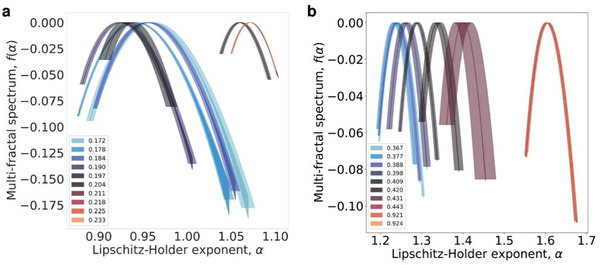
Node‐based multifractal analysis. The multifractal spectra of OTAB in the range for different molar fractions corresponding to a) micellar to hexagonal and b) hexagonal to lamellar phase transitions. Each node in the graph is considered as the origin of a growing box. As the box size increases, the fractality manifests as an exponential growth of the number of nodes covered by the box. Then, the multifractality of the graph can be characterized by the corresponding spectrum obtained by the Legendre transform.

The strong shifts in the Lipschitz‐Hölder exponent are observed for the transition from micellar to hexagonal phase (*x* = 0.204, **Figure** **8**a). As expected, the formation of the cylinders results in considerable narrowing of the multifractal spectrum *f(α)* due to the emergence of the repeatable structural pattern. Simultaneously, the absolute values of *α* increase because of the organization of the phases both at molecular and nanometer scale. Also, the increased order in the molecular packing in the hexagonal phase leads to long‐range interconnectivity with greater divergence in connectivity patterns across different scales (Figures 5a,b and 6a,b), therefore resulting in larger *α* corresponding to higher network complexity. Nearly identical behavior is observed for the phase transition between hexagonal and lamellar phases (Figure 8b) with the exception that *f(α)* is already quite narrow and does not change too much. However, the formation of the lamellar phase is associated with the formation of the nanosheets with even larger physical dimensions than cylindrical micelles, shifting multifractal spectrum to even greater values of *α*.

## 3 Conclusions

GT and multifractality descriptors can successfully identify phase transitions involving complex molecular architectures and characterize the pathways between them. Degree centrality, clustering coefficient and NFD vividly distinguish between five types of surfactant aggregates (Figures S7 and S8, Supporting Information) and identify different long‐range patterns, collective interactions, and structural fluctuations in a given phase occurring during phase transitions. Besides liquid crystals and surfactants, this methodology can be generalized for other (supra)molecules and particles capable of self‐organization into complex structures with organizational patterns at multiple scales by following a protocol similar to the one employed here: structural information may be obtained from either computer simulations or microscopy experiments, and the definition of a convenient cutoff distance defines which molecules, atoms, ions, aminoacid residues or nanoparticles (the nodes of the graph) should be considered as neighbors and, as such, should be connected by an edge in the graph. Phase transitions which result in more interconnected structures should present a sudden increase in closeness centrality values as observed here in the micellar to hexagonal and in hexagonal to lamellar transitions. The betweenness centrality, on the other hand, will increase if after a phase transition, the removal of a few molecules or particles can disrupt the network between several particles, as in the cylinders of the hexagonal phase, and decrease if the network becomes less sensitive to the removal of a few particles as in the lamellar phase. The success in applying those metrics will of course depend on the criteria used to define which particles should be considered neighbors, which may be improved by adding other criteria besides a simple cutoff as used here, like the relative orientation between consecutive nodes for the study of a system involving hydrogen bonds or oriented dipoles. One of the exciting future directions will be the description of phases in mixtures of surfactants or complex salts^[^ ^50^ ^]^ with nanomaterials, such as graphene,^[^ ^51^, ^52^ ^]^ and nanoparticles. Multifractal graph characterization of different structural patterns inside a given phase may be used to quantify, understand, and engineer the phase transition pathways for a given change in experimental conditions, for the templated assembly and synthesis of hierarchical nanomaterials.^[^ ^53^ ^]^

## 4 Experimental Section

### Molecular Dynamics

All molecular dynamics simulations described here were performed with Gromacs 2018.4 package^[^ ^54^, ^55^ ^]^ and the resulting trajectories were visualized with VMD 1.9.3 software, which was also used to render the graphical representations shown here.^[^ ^56^ ^]^ Specific details of system preparation and simulation conditions are given below for each model system.

### Argon Simulations

To study how graph theory can describe the solid‐liquid transition, simulations of Argon were performed at different temperatures starting from a FCC crystal with 2916 argon atoms with the initial distances between neighbor atoms computed based on the position of the minimum of the Lennard‐Jones potential between argon atoms.^[^ ^57^ ^]^ An initial relaxation of the crystal was performed at 20 K and then simulations were performed at *T* = 60, 70, 75, 80, 85, 90, 95, 100, 105 and 110 K, with the simulation at each temperature starting from the final structure of the simulation at the previous temperature. Berendsen thermostat and barostat were used for all simulations with *τ*_T_ = 0.1 ps, *P* = 1 bar and *τ*_P_ = 1.0 ps. A cutoff radius of 1.2 nm was employed for the Lennard‐Jones potential with a shift function. A timestep of 0.001 ps was employed in all simulations with each one reaching a total integration time of 5 ns.

### Liquid Crystal Simulations

In order to study temperature‐driven phase transitions in a liquid crystal, a model system was prepared by randomly placing 3200 1‐hexadecyl‐3‐metilimidazolium cations and 3200 tetrafluoroborate anions in a cubic simulation box with 14.0 nm edges using the Packmol software.^[^ ^58^ ^]^ The model system was simulated at the temperature of 320 K and pressure of 1 bar for 500 ns at which the spontaneous reorganization from the isotropic to the anisotropic liquid crystal structure was observed. 500 ns simulations at *T* = 340, 360, 380, 400, 420, 440, and 460 K were performed with each one starting from the final structure of the T = 320K simulation. To verify how graph theory metrics can capture hysteresis in phase transitions, in addition to the simulations at constant temperatures, starting from the same initial structure, 240 ns simulations were performed at which the system was submitted to 3 heating and cooling cycles where the temperature of the external bath was first increased by 40 K at a constant rate of 1 K per ns and then reduced to the original temperature also with a constant rate of 1 K per ns. Three simulations were performed with the heating/cooling cycles with the temperature ranging from 340 to 380 K, 380 to 420 K, and 420 to 460 K. Only the range between 380 and 420 K captures the phase transition, with the other temperature ranges performed to study how metrics responds to heating and cooling in absence of the transition.

All those simulations were performed with Martini 3.0 force field^[^ ^59^ ^]^ with V‐rescale algorithm^[^ ^60^ ^]^ for temperature coupling with *τ*_T_ = 1.0 ps and Berendsen weak coupling for pressure with *P* = 1 bar and *τ*_P_ = 1.0 ps. A cutoff radius of 1.1 nm was employed for both coulomb and Lennard‐Jones potential with particle‐mesh Ewald (PME)^[^ ^61^ ^]^ long‐range corrections and a relative dielectric constant of 15 for the electrostatic interactions and a shift function was used to ensure the Lennard‐Jones potential goes smoothly to zero at the cut‐off. An integration timestep of 0.02 ps was employed in all simulations.

### Water/Surfactant Simulations

The MD simulation of surfactant systems is computationally too demanding both in the low concentration regime, where the large mean free path limits the ability of surfactant molecules to sample their phase space, and in the high concentration regime, where progressively more rigid lattices drastically reduce the diffusion of the molecules, also limiting their ability to sample the available phase space. These intrinsic limitations are less severe for short‐chain surfactants, since the molecular relaxation times of the systems increase exponentially with the size of the hydrophobic chain,^[^ ^29^ ^]^ and for coarse‐grained potential energy surfaces, which decrease the size of the available phase space, allowing a more efficient sampling of microstates. These two requirements were met by choosing the surfactant *n*‐octyltrimethylammonium bromide (OTAB) and the Martini 3.0 coarse grained force field^[^ ^58^ ^]^ with the same cutoff radius and long‐range corrections described for the liquid crystal. On this force field, the surfactant is described by a polar head bead with interaction type Q2, charge 1.0 e and mass 59.13 g mol^−1^ and two hydrophobic beads used to describe the 8 carbon tail with type C1, charge 0 and masses 56.12 (middle bead) and 57.13 g mol^−1^ (terminal bead). The bromide counter‐ions are described by a single bead with type SQ4, charge −1.0 e and mass 79,90 g mol^−1^ and the water is described by an equimolar mixture of the neutral beads W, SW, and TW, which presents different sites and represents clusters of 2, 3, and 4 water molecules each. The mixture of those sites consist in an essentially ideal solution to describe the solvent but add more flexibility to the CG model.

The initial structure was constructed by randomly placing 800 OTA^+^ cations, 800 Br⁻ counter‐ions and 117600 water particles (which corresponds to 352800 water molecules) in a cubic box with edge length of 25 nm using the software Packmol.^[^ ^57^ ^]^ The energy of the system was minimized using the steepest descent algorithm and a 1000 ns MD simulation was performed to equilibrate the model system, spontaneously yielding a stable suspension of several nearly spherical micelles and decreasing the edge length of the cubic box to 22.04 nm. The phase space was sampled in the constant‐NPT ensemble increasing the surfactant concentration stepwise from the initial molar fraction of *x* = 0.002256 by the removal of water particles. At each step along the phase diagram, 4% of the remaining amount of water particles plus six additional particles were randomly selected and removed from the system, followed by 30 ns of MD simulation to relax the system to its new position in the phase diagram. The first 15 ns of each MD simulation were discarded to ensure that only relaxed structures were analyzed except in data of Figures 5 and 6, which focus in the fluctuations that happens before the transitions. This protocol removes larger amounts of water molecules at the dilute regime as compared to the more concentrated solutions, favoring the sampling of the later, where the phase transitions of interest take place, while the former is a large region of the phase diagram characterized by continuous structural changes instead of actual phase transitions. A total of 170 simulations were performed until the complete removal of the solvent, with a total simulation time of ca. 5 µs (the composition of all model systems may be found in Table S1, Supporting Information). In order to evaluate the effect of the size of the model system in the GT metrics, 120 ns simulations were also performed with 8 times larger systems (6400 surfactant ions) for which the initial structure was created duplicating the final structure of the original simulation at the same molar fraction.

For comparison purposes, additional simulations were performed for ideal solutions of the OTAB in water. These simulations were performed starting from the final structure at each composition along with the phase diagram scan but performing a NVT simulation where the intermolecular interaction parameters for both surfactant and bromide sites were changed to render a nearly ideal mixture with water with no tendency to aggregation as shown by RDF in Figure 3b and the structures and graphs in Figures S1 and S2 (Supporting Information).

The dependence of the GT descriptors on the size of the model system was evaluated by comparing the data from MD simulations using a model system eight‐fold larger than the standard one. The phases, their key GT descriptors, and their trends with OTAB molar fraction remained the same for both small and large models (Figure S9, Supporting Information). The absolute value of closeness centrality decreased with the increase in the system size as expected since it is computed based on the node‐to‐node distance, which increases with the graph size.

Every OTAB system simulation were performed at NPT ensemble with *T* = 300 K and *P* = 1 bar hold with Berendesen coupling scheme with *τ*_T_ = 1.0 ps and *τ*_P_ = 0.5 ps except for ideal solutions, which were simulated at constant volume instead of constant pressure, holding the same density of the real solution in the same molar fraction. The use of coarse grained models enables the use of the relatively large integration timestep of 0.02 ps in every simulation presented here since the masses of the sites are much larger than typical atomic masses and the force constants of harmonic potentials between sites are much smaller than those in atomistic models, so no high frequency vibrational normal mode is present in the model. Periodic boundary conditions were employed in all directions.

### Free Energy Calculations (Alchemical Simulations)

In an alchemical transformation a chemical species *A* is transformed into another species *B* through “unphysical” (alchemical) intermediates. Free energy estimates can be made by making use of the fact that the free energy is a state function and as such it does not depend on the path taken even if the path contains such unphysical structures. The alchemical free energy can be estimated from either equilibrium or nonequilibrium simulations, and the equilibrium case is focused. In practice, the equilibrium alchemical free energy calculations are performed using multistates equilibrium simulations connecting a set of state *A* to state *B*.

The differences between two states are related to the ratio of probabilities between those two states. Because the phase space overlap between *A* and B is usually near zero, the direct *A* → *B* free energy would lead to unreliable results. To improve the results, the thermodynamic path can be reconstructed, including high phase space overlap intermediates and calculate the free energy as

(1) $$\Delta G=k_{B}\;T\sum_{k\;=\;0}^{K-1}\Delta f\left(\lambda_{k},\lambda_{k+1}\right)$$

from samples at all alchemical states $\left\{\vec{\lambda }\right\}$ and Δ*f* is the unitless free energy difference Δ*f* (λ_*k*_,λ_*k* + 1_) =  Δ*f*(λ_*k* + 1_) − Δ*f* (λ_*k*_) =   − *lnQ*(λ_*k* + 1_)/*Q*(λ_*k*_).

Free energy is a state function, then the alchemical potential can be used in two end points Hamiltonians (ℋ) from molecular dynamics simulations,

(2) $$H\left(\lambda \right)=\lambda H_{B}+\left(1-\lambda \right)H_{A}$$

For this application, the momentum is disregarded because neither the number of particles nor the total mass of system changes, then

(3) $$H\left(\lambda \right)=\;\lambda H_{B}+\left(1-\lambda \right)H_{A}$$

where *H* is the enthalpy and λ ranges from 0 to 1.

The free energy calculations were performed with 43 λ windows (0.00, 0.03, 0.05, 0.07, 0.10, 0.13, 0.15, 0.17, 0.20, 0.23, 0.25, 0.27, 0.30, 0.33, 0.35, 0.37, 0.40, 0.42, 0.45, 0.47, 0.50, 0.53, 0.55, 0.58, 0.60, 0.63, 0.65, 0.67, 0.70, 0.73, 0.75, 0.77, 0.80, 0.83, 0.85, 0.87, 0.90, 0.915, 0.93, 0.95, 0.97, 0.985, 1.00). The last frame of the equilibrium MD simulations served as the starting configuration for the NPT windowed calculations, performing 30 ns MD simulations to relax the structure at each window, followed by 20 ns MD production simulations (summing over 43 * 20 ns = 860 ns), for each free energy calculation. The production simulations were carried out with Parrinello‐Rahman coupling scheme with τ_T_ = 20.0 ps and Nose‐Hoover thermostat with τ_T_ = 10.0 ps.

### Idealized Structures

In order to better understand how network metrics are related to possible structures in surfactants systems, five idealized structures of OTAB in water were construct and simulated. None of those structure are expected to be stable in dilute aqueos solution for this surfactant and harmonic potentials were used to hold the structure stable while still enabling some flexibility as described below.

### Bidimensional Micelle

A cluster of 8 OTA^+^ cations where draw with every site with the same z coordinate and with hidrophobic sites pointing toward the center of the cluster. An harmonic potential with a force constant of 2500 kJ mol^−1^ nm^−2^ was applied over the z coordinate of every site to enable only small out of plane vibrations while holding the cluster essentially bidimensional. A weaker force constant of 100 kJ mol^−1^ nm^−2^ was applied between the terminal tail site and the cluster center.

### Infinity Cylinder

Produced by stacking up 16 bidimensional micelles described above along the z coordinate. The restraint over the z coordinate was removed so the surfactants can diffuse along z axis but the constraint over the terminal tail sites of 100 kJ mol^−1^ nm^−2^ was still applied in the xy plane in order to avoid dissociation and the break of the structure into micelles. Due to periodic boundary conditions, the surfactants in the bottom are in contact with the ones on the top of the structure, thus creating an infinity cylinder.

### Monolayer

A layer of 224 OTA^+^ cations with hexagonal arrangement was constructed with all cations perpendicular to the *z* direction with the same orientation and the head site with the *z* value. Force constants of 175 kJ mol^−1^ nm^−2^ and 17.5 kJ mol^−1^ nm^−2^ were applied to constrains head and tail sites, respectively, along *z* direction.

### Bilayer 1

Produced exactly like the monolayer but with half of OTA^+^ cations pointing in the opposite direction, which renders two hydrophilic surfaces instead of a single one. The constraints are the same as in monolayer.

### Bilayer 2

Produced exactly like monolayer but including a second layer of surfactant with opposite orientation and placed with the hydrophobic tails in contact with the first layer. The constraints are the same as in monolayer.

The structures went initially to an energy minimization freezing the surfactant sites in the same directions the contraints reported above were used. For the monolayer system, a relaxation with stronger force constants were also performed before the MD with the constraints reported above since even with those force force constants the monolayer partially break up due to intense repulsion between surfactant heads if the counterions are not adsorbed yet. 20 ns simulations were performed for each system with the last 10 ns used for the analyses. Semiisotropic pressure coupling was used with the box dimensions held constant in X and Y directions and enabling only changes in the Z dimension. Other conditions were the same as described for phase diagram scan.

### Graph Visualization

Graph visualizations of the corresponding snapshots (for example in Figure 5b) are generated using NetworkX with Fruchterman‐Reingold force‐directed algorithm, which treats node as force and edge as spring to physically simulate the positions.^[^ ^62^ ^]^ While there is a distinct relationship between the structure of the phase and the morphology of the graph, clustering of the nodes in the Fruchterman‐Reingold graphs does not necessarily represent the clustering of the particles in physical space.

### GT Descriptions of the MD Models

To mine the higher‐order topological statistics of phase transitions of OTAB, various GT metrics of the structures are estimated along the phase diagram using the distance between surfactant tail sites as a criterion to create the graphs. Since the progression toward a phase transition may manifest through the emergence of multiscale long‐range topological correlations, how GT metrics are capable of capturing *k*‐th point distributions (that can be viewed as counterparts of the higher‐order statistics from probability theory) is investigated and evolved as the molecular graph approaches the phase transition. Towards this end, the following GT order parameters are considered.

### Degree Centrality

Degree centrality of a node represents the degree of that node normalized by the maximum possible degree in the graph.^[^ ^48^ ^]^ It measures the fraction of connected nodes in the whole graph and is calculated as:

(4) $$C_{d}\left(v\right)=\frac{\deg\left(v\right)}{N-1}$$

where deg(*v*) is the degree of node *v*.

### Closeness Centrality

Closeness centrality measures how close the node is to other nodes in topological space; it is calculated using the sum of all shortest paths from the node to other nodes.^[^ ^43^ ^]^ The closeness centrality with Wasserman and Faust improved formula^[^ ^63^ ^]^ of a node *v* is calculated as follows:

(5) $$C_{c}\left(v\right)=\frac{N_{v}\vee -1}{N-1}\frac{N_{v}\vee -1}{\sum_{u\in N_{v}}d\left(v,u\right)}$$

where *N_v_* are the set of reachable nodes to the node *v*, *N* denotes the total number of nodes in the system, *N_v_* ∨ represents the number of node *v*’s neighbors, and *d*(*v*, *u*) is the shortest path distance between nodes *v* and *u*, respectively.

### Betweenness Centrality

Betweenness centrality represents how often a given node is the shortest path between other two nodes.^[^ ^43^ ^]^ The betweenness centrality of a node *v* is computed as:

(6) $$C_{b}\left(v\right)=\sum_{s,t\in V}\frac{\sigma_{s,t}\left(v\right)}{\sigma_{s,t}}$$

where *V* is the set of nodes in the graph, σ_*s*,*t*_ is the number of shortest path from node *s* to *t*, σ_*s*,*t*_(*v*) is the number of those shortest paths passing through node *v*.

### Clustering Coefficient

Clustering coefficient quantifies the degree to which the nodes tend to cluster together.^[^ ^48^ ^]^ The clustering coefficient of node *v* in a binary graph is calculated as follows:

(7) $$\mathrm{CC}\;\left(v\right)=\frac{T\left(v\right)}{\deg\left(v\right)\left(\deg\left(v\right)-1\right)}$$

where *T*(*v*) is the number of triangles in the graph that contain node *v*.

### Multifractal Topological Analysis: Node‐Based Fractal Dimension (NFD)

This descriptor measures the self‐similarity of a graph by calculating the spatial dimension of the expansion from a certain node based on the box‐growing method.^[^ ^44^ ^]^ Let us consider the node *i* in the graph as the origin of the box. the box radius is increased to *r*, and the number of nodes in the box as *M*(*r*) is recorded, then the relationship between *M*(*r*) and *r* can be characterized as: *M*(*r*) ∼ *r^d^*, where *d* is the NFD, capturing the generating rule of the graph with a certain node as the origin of the graph.

### Node‐Based Multifractal Analysis (NMFA)

NMFA is a method to quantify the multiple topological generating rules and fractal properties of graphs across multiple topological scales. Based on the above‐mentioned fractal dimension concept, to distinguish the multiple generating rules of different nodes in the graph, the distorting exponent *q* is introduced. The partition function *U_q_* (*r*) is defined as:

(8) $$U_{q}\;\left(r\right)=\sum_{1}^{N}{\left(\frac{M_{i}\left(r\right)}{N}\right)}^{q}$$

where *M_i_* (*r*) is the mass distribution of the original node *i* when the box radius is *r* (i.e., the number of nodes that are covered by the box of radius *r* originating from node *i*), and *N* is the total number of nodes in the graph. The mass exponent τ(*q*) is used to capture the power law relationship between *U_q_* (*r*) and *r*:

(9) $$\tau \;\left(q\right)=\frac{\ln\left(U_{q}\left(r\right)\right)}{\ln\left(r\right)}$$

By exploiting the Legendre transform, the multiple generating rules of the graph can be estimated by the multi‐fractal spectrum*f*(α):

(10) $$\alpha =\frac{d\tau \left(q\right)}{dq}$$

(11) $$f\left(\alpha \right)=q\alpha -\tau \left(q\right)$$

where α is the Lipschitz‐Holder exponent measuring the complexity of the fractal structure in the graph. In general, a higher value of α represents a higher degree of complexity. In the node‐based multi‐fractal analysis, the multi‐fractal spectrum shows the distribution of multiple generating rules of the graph. The Lipschitz‐Holder exponent α quantifies the degree of complexity of the graph structure and the width of the spectrum (*w*  = α_max_  − α_min_) quantifies the degree of heterogeneity.

### Simple Example Provided for NFD and NMFA

In order to better understand the topological meaning of NFD and degree of complexity and heterogeneity quantified by NMFA, the typical graph example is provided (i.e., Sierpinski fractal graph) here. Figure S10 (Supporting Information) shows the box‐growing method on the Sierpinski fractal graph with different generating rules: *b*  =  3, *f*  = 1/3 ;*b*  =  6, *f*  = 1/3 . The monofractal growth rules can be also characterized by NFD, capturing the power law relationship between the box size and the number of nodes in the box, that is, NFD  =  ln(*b*)/ln(1/*f*). The monofractal graph has a single generating rule, displayed as a node on the multifractal spectrum. The higher NFD value representing the more complex generating rule will bring a higher Lipschitz–Holder exponent value on the multifractal spectrum, which indicates the higher degree of complexity. For the multifractal graph with multiple generating rules, the multifractal spectrum shows the distribution of the rules. Therefore, the width of the spectrum characterizes the diversity of the generating rules or the degree of heterogeneity.

## Conflict of Interest

The authors declare no conflict of interest.

## Author Contributions

R.Y. and K.B. contributed equally to this work. R.Y. and K.B.—drawing model systems, running simulations, performing analyses, and writing original draft; X.X., W.R.G., and D.A.M.—running simulations and performing analyses; N.A.K., P.B., and A.F.M.—conceptualization, reviewing and editing.

## Supporting information

Supporting Information

## Data Availability

The data that support the findings of this study are available from the corresponding author upon reasonable request.
