# Supplementary material for: Graph Theoretical Description of Phase Transitions in Complex Multiscale Phases with Supramolecular Assemblies
Source: Adv Sci (Weinh). 2024 Jul 1;11(33):2402464. doi: 10.1002/advs.202402464 (PMC11967988; doi:10.1002/advs.202402464)
Supplement: Supplementary file 1 — Supporting Information [file ADVS-11-2402464-s001.pdf]

ADVANCED SCIENCE
Open Access

Supporting Information

for *Adv. Sci.*, DOI 10.1002/advs.202402464

Graph Theoretical Description of Phase Transitions in Complex Multiscale Phases with Supramolecular Assemblies

Ruochen Yang, Kalil Bernardino, Xiongye Xiao, Weverson R. Gomes, Davi A. Mattoso, Nicholas A. Kotov, Paul Bogdan* and André F. de Moura**

Supplementary Information

Graph theoretical description of phase transitions in complex multiscale phases with supramolecular assemblies

Ruochen Yang,[1,2†] Kalil Bernardino,[3†] Xiongye Xiao,[1,2] Weverson R. Gomes,[3] Davi A. Mattoso,[3] Nicholas A. Kotov,[2,4*] Paul Bogdan[1,2*] and André F. de Moura[3*]

[1] Ming Hsieh Department of Electrical and Computer Engineering, University of Southern California, Los Angeles, CA, 90089, USA.

[2] Center of Complex Particle Systems (COMPASS), USA;

[3] Department of Chemistry, Federal University of São Carlos, São Carlos, SP, 13565-905, Brazil.

[4] Department of Chemical Engineering, Department of Materials Science and Engineering, Biointerfaces Institute, University of Michigan, Ann Arbor, MI, 48109-2102, USA.

[†] These authors contributed equally.

[*] Corresponding authors: kotov@umich.edu, pbogdan@usc.edu and moura@ufscar.br

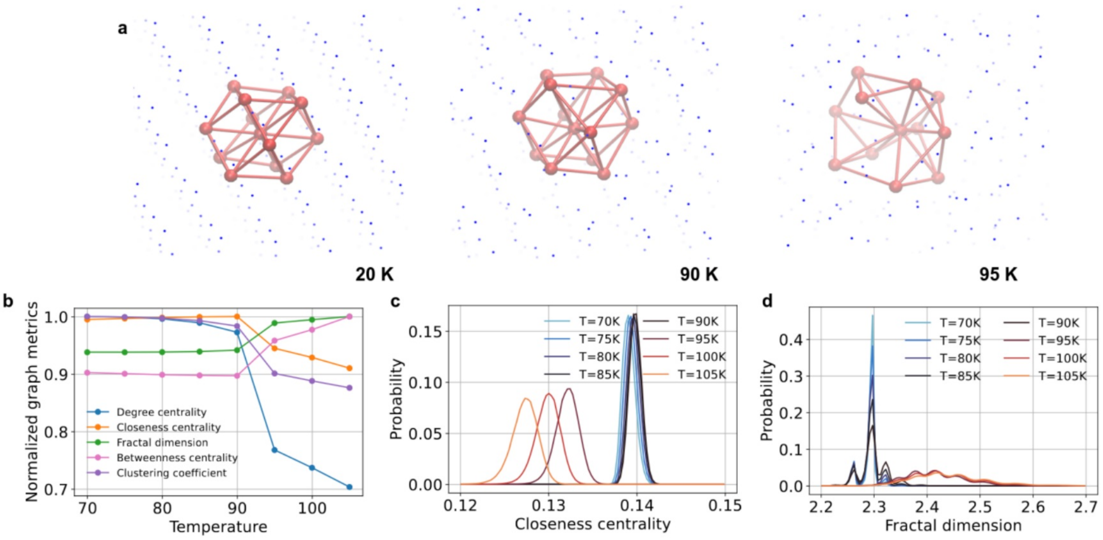

Fig. S1. Additional structures and graph metrics analyses for argon solid-liquid transition. **(a)** Final structure of solid argon simulations at 20 K and 90 K, and of liquid argon at 95 K, highlighting one randomly selected atom and its closest neighbors as red spheres and including bonds between them if the distance is smaller than 0.49 nm and a color scale was applied só atoms closer to the reader are displayed in darker colors than the atoms farther to the reader. **(b)** Average values of several GT metrics computed in the simulations of argon at different temperatures with the values normalized in order to the highest value for each metric being equal to 1. **(c)** and **(d)** distributions of closeness centrality and node based fractal dimension at each temperature.

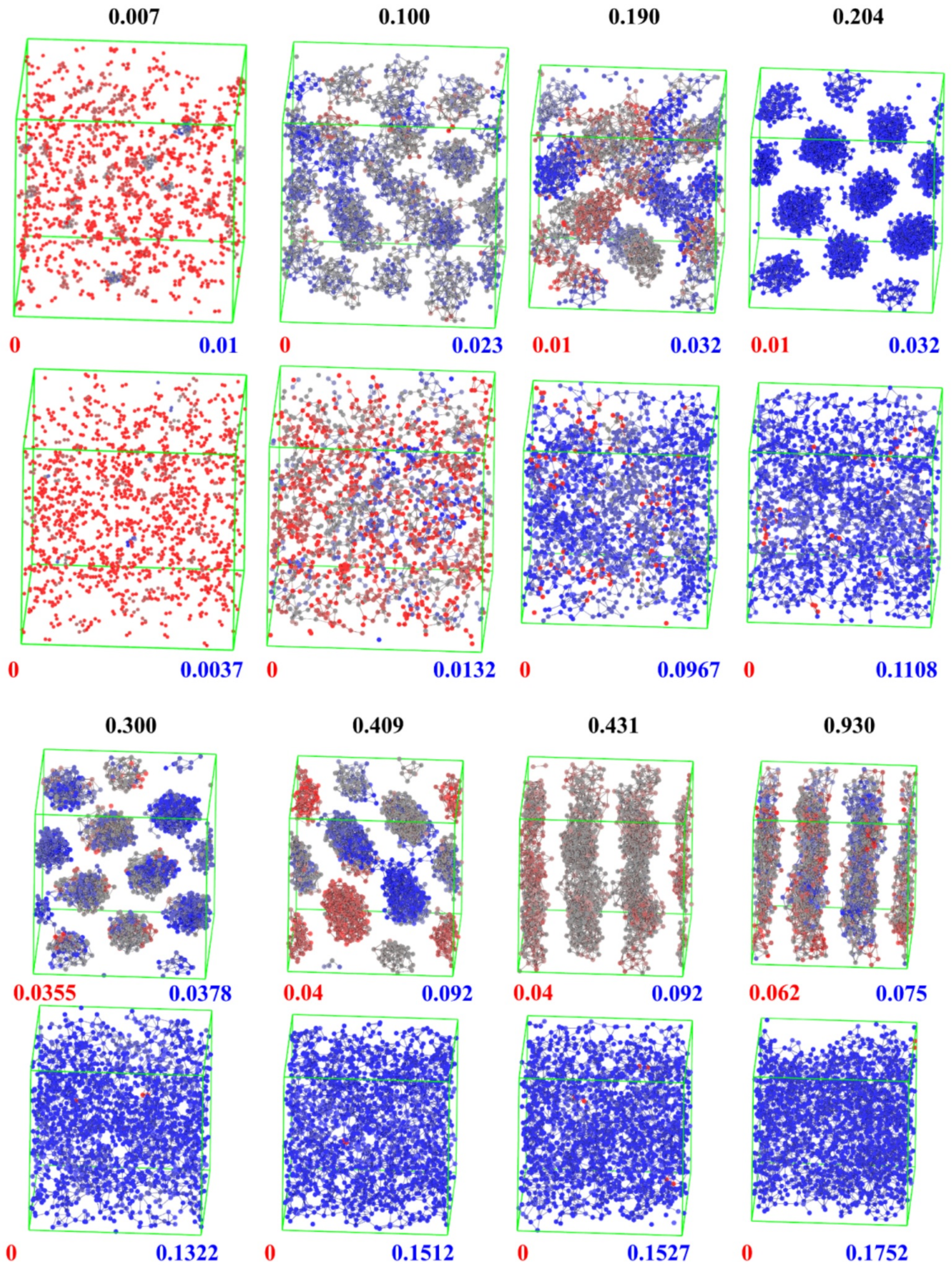

Fig. S2. Graphical representations of the structures from which the graphs of **Fig. S2** were computed showing only surfactant tail sites colored using closeness centrality values (range of values used to color given in the bottom of each structure). Surfactant molar fraction is given by black numbers above structures. For each molar fraction, the structure in the top corresponds to the real solution with the one below corresponds to the ideal solution. Connections were drawn between tail sites within 0.7 nm from each other, the same cut radius used to define the connections in the construction of the graphs. Green lines indicate the boundaries of the simulation box, which is shown in the same scale for every composition except x = 0.007, so the shrinking in box size with composition reflect the actual reduction of the simulation box for mole fractions larger than 0.1.

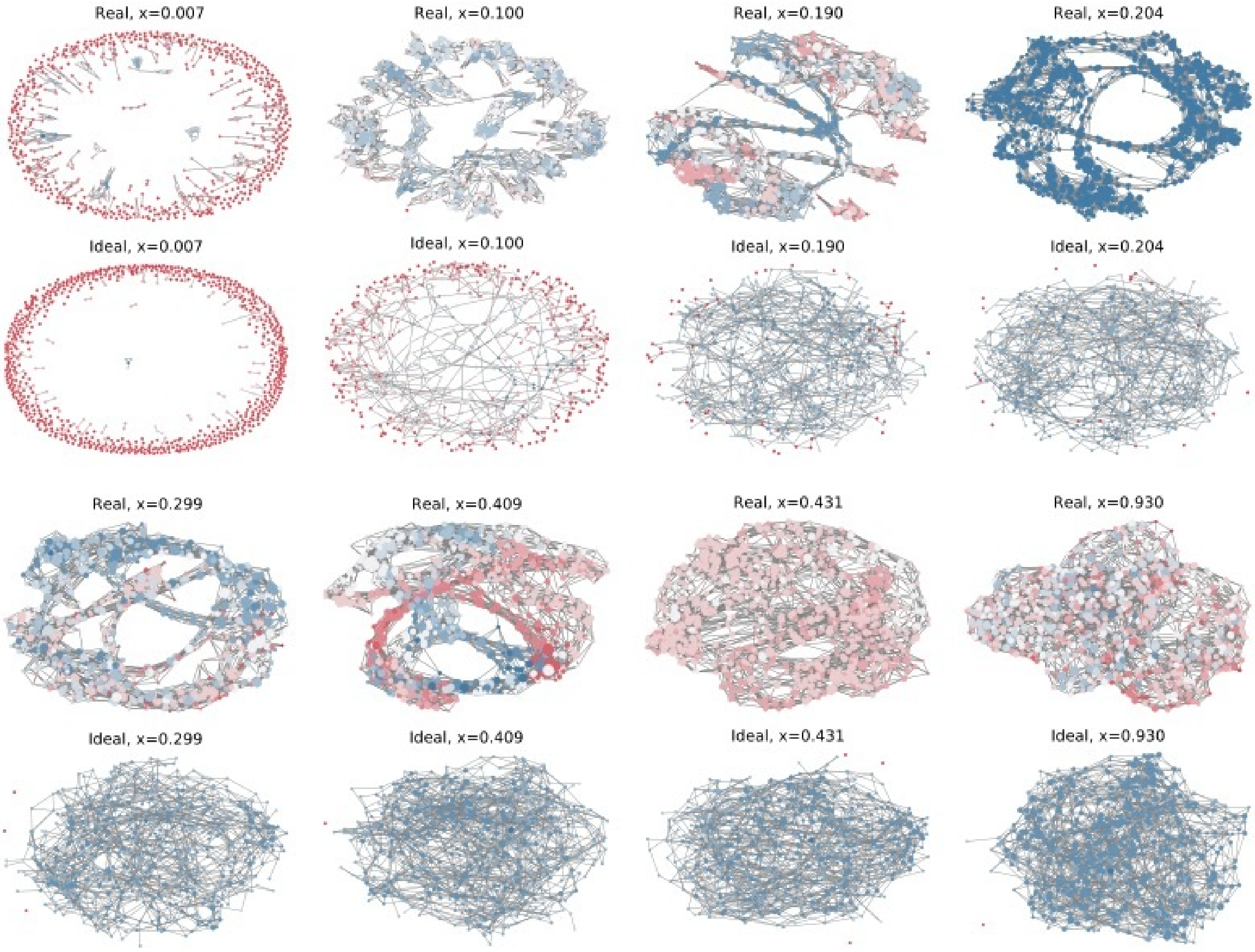

Fig. S3. Graph visualization for the same structures from MD simulation shown in **Fig. S2**. Nodes were colored based on closeness centrality value using the same color scale as **Fig. S2**.

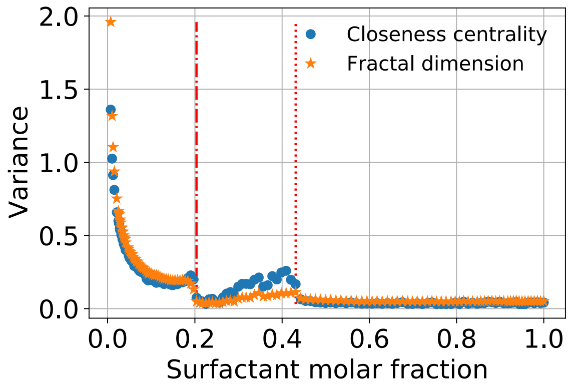

Fig. S4. Variance of the closeness centrality and node-based fractal dimension (NFD) during the molecular dynamic simulation. A significant drop of the variation of both closeness centrality and fractal dimension (NFD) at molar fraction $x = 0.204$ (transition from micelle to hexagonal) can further support our observation of the phase transition.

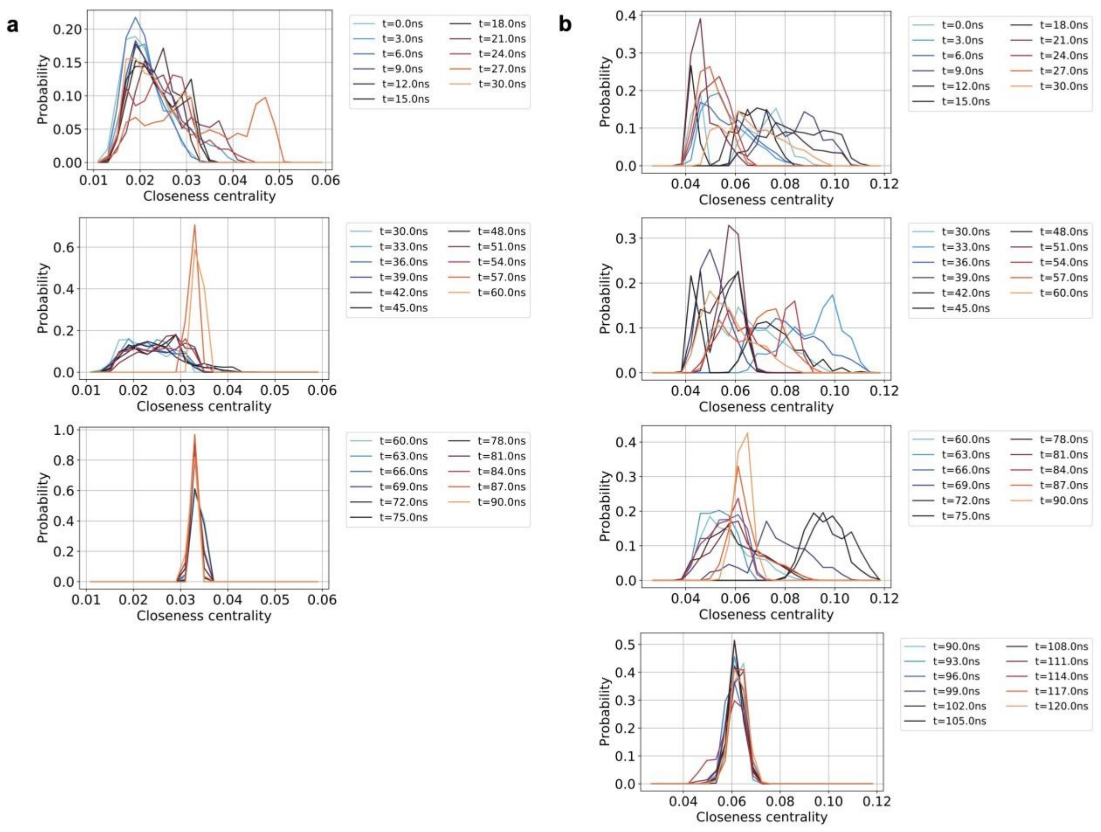

Fig. S5. Closeness centrality distribution of **(a)** the selected snapshots of **Fig. 3** around phase transitions from micelle to hexagonal and **(b)** around the transition from hexagonal to lamellar in **Fig. 4**.

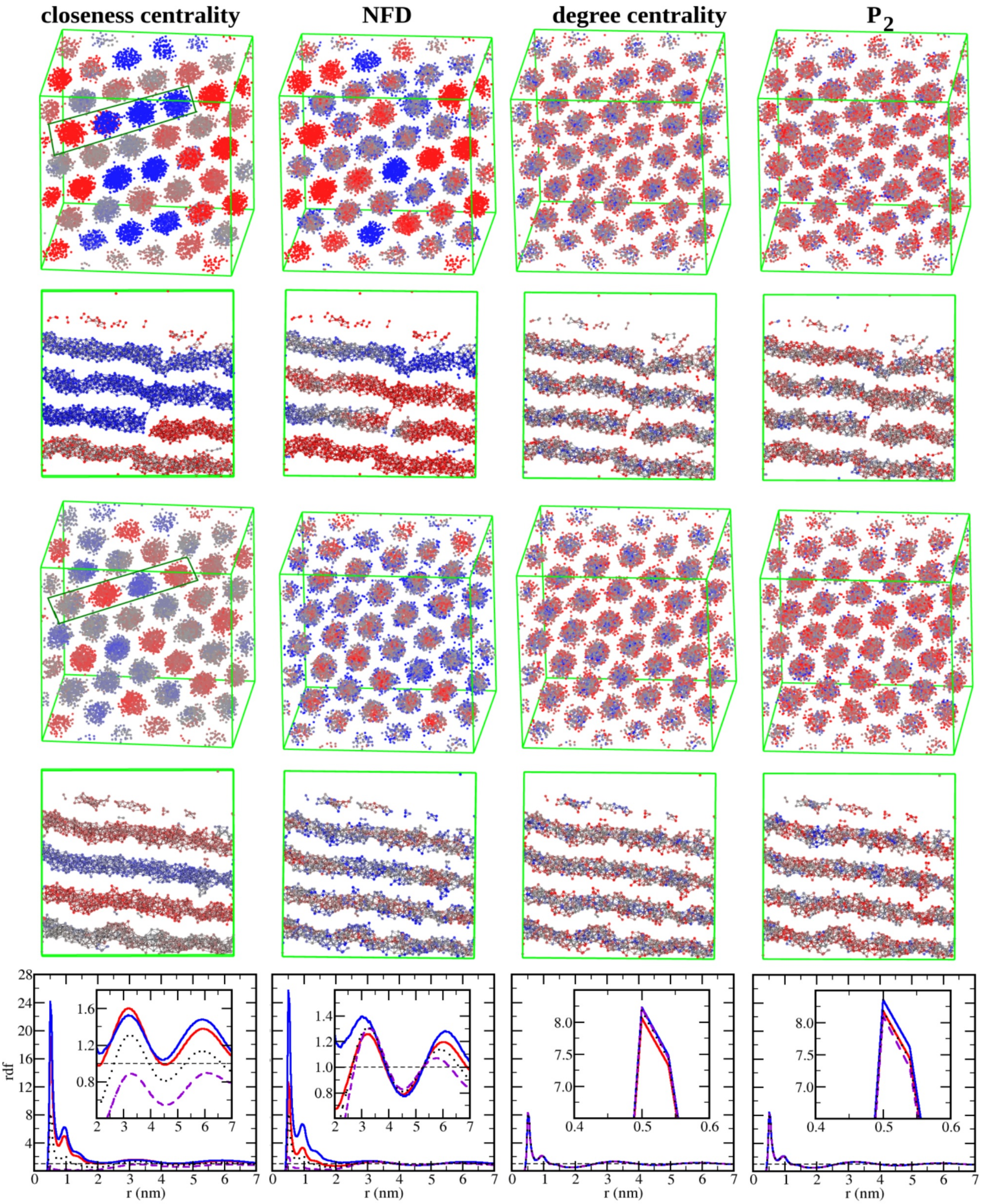

Fig. S6. Two selected structures for the larger model system with the OTAB molar fraction of 0.2177 showing only the surfactant tail sites colored using (from left to right) values of closeness centrality, NFD, degree centrality and P_2, with red and blue colors representing low and high values of the respective metric. Below the lateral view of the whole system, another representation of the same structure is given focusing on the lateral view of four tube segments marked with the dark green lines on the left, in which lines were included to highlighted the surfactant tails that are connected in the construction of the graph. At the

bottom is shown the radial distribution function between the terminal tail site of surfactant molecules with red curves including only the 25% molecules with lowest values for the metric, blue curves including only the 25% with highest values, purple dashed curves gives the distribution of the 25% higher in relation to the 25% lower and the dotted black curves include every surfactant molecule. Insets zoom over the long-range structure for closeness centrality and fractal dimension and over the first peak for other metrics.

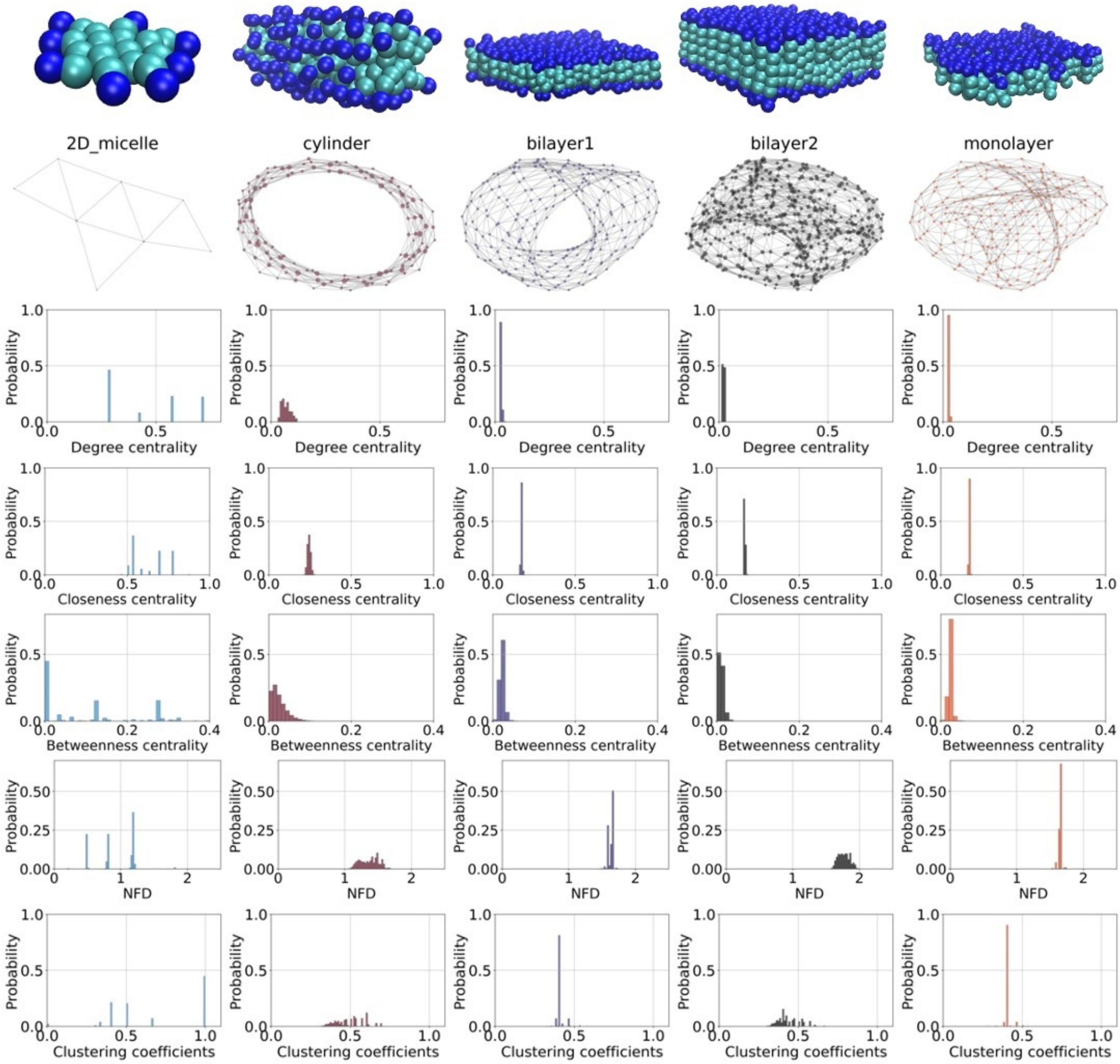

Fig. S7. GT descriptors, *i.e.*, closeness centrality, degree centrality, betweenness centrality and clustering coefficients, calculated for five idealized systems (bidimensional micelle, bilayer 1, bilayer 2, infinity cylinder micelle and monolayer) using the terminal tail sites as nodes.

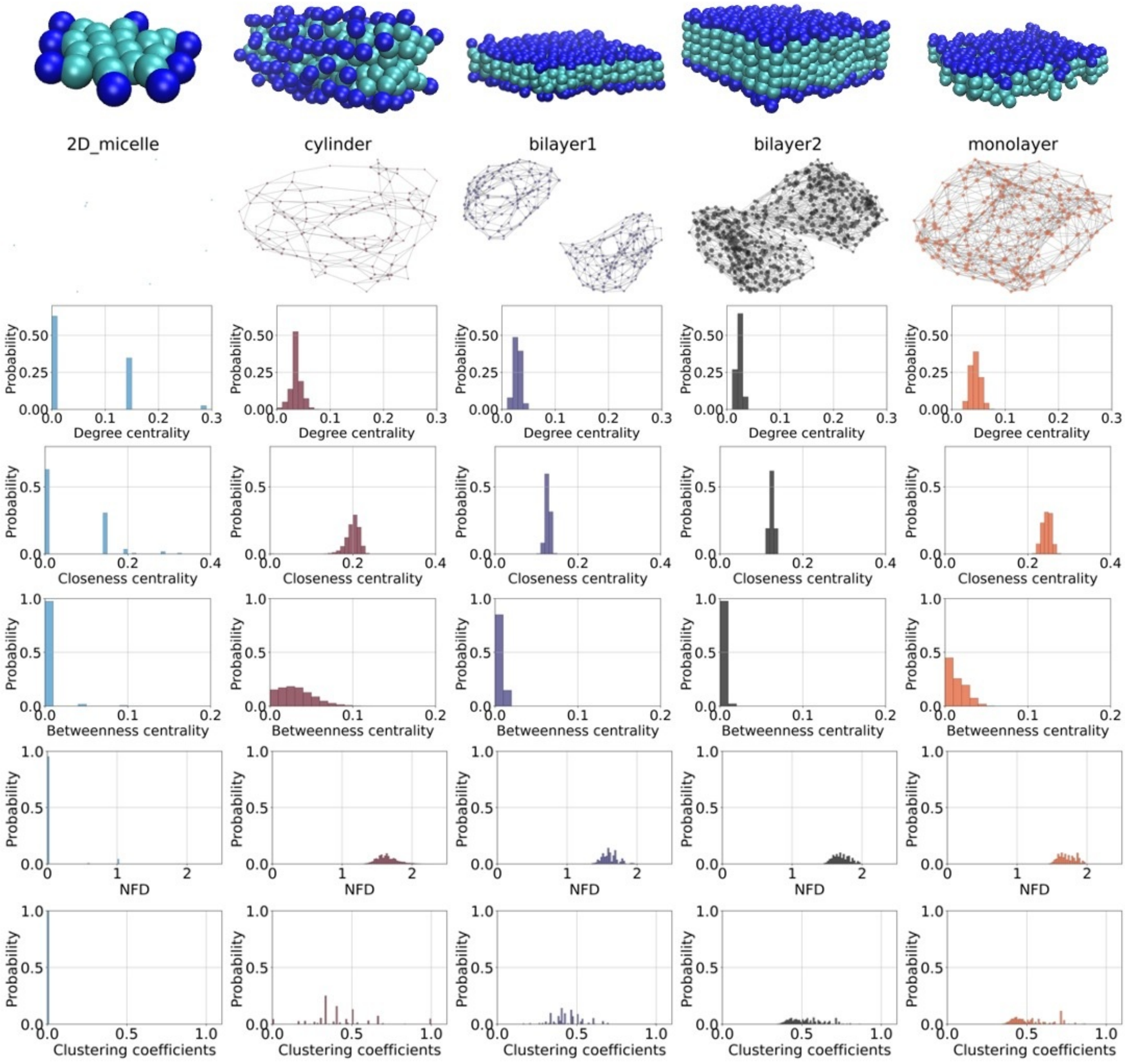

Fig. S8. Graph properties (closeness centrality, degree centrality, betweenness centrality and clustering coefficients) calculated for the same five idealized systems in **Fig. S6** using the head sites as nodes to build network.

Supplementary Comment: We also considered the GT metrics for five idealized systems: bi-dimensional micelles, infinite cylinders, monolayers and two distinct bilayers, one with the surfactant tails interdigitated (bilayer 1) and the bilayer formed by two dense monolayers (bilayer 2). Although these structures have not been experimentally observed for OTAB, these simulations allow one to test the ability of GT to distinguish between these different organizational patterns (**Fig. S7**). Descriptors, such as degree centrality, clustering coefficient and nodal fractal dimension, can distinguish between these structures, showing different average values and/or sharpness of the distribution. The cases of surfactant molecules organized in

the nearly identical structures, such as bilayer 1 and monolayer, yield similar results for every metric concerning the tail-tail contacts. If the same metrics are considered for the head-head contact, then a clear distinction between those two structures also emerges (**Supplementary Fig. 8**).

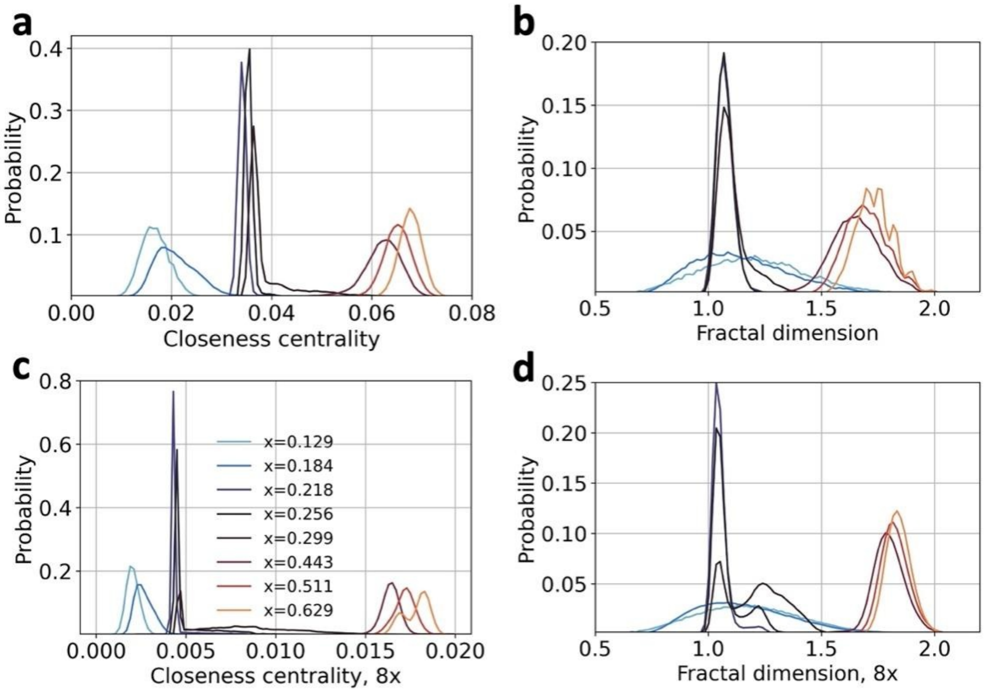

Fig. S9. Distribution of values of closeness centrality (left) and fractal dimension (right) for selected compositions of the original model systems (top) and eight times larger model systems (bottom).

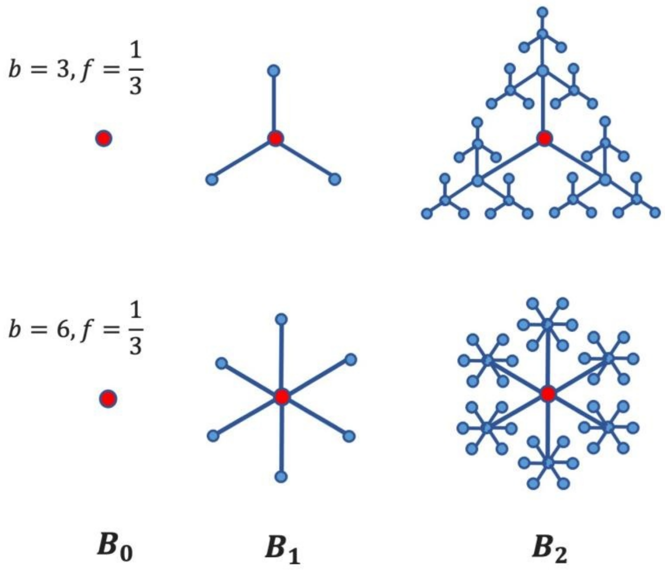

Fig. S10. The box-growing method on the Sierpinski fractal graphs under different copy factors b and a scaling factor f.

Table S1 - Number of water particles, N_W*, surfactant molar fraction, x_{mol}, and mass fraction, x_{mass}, in every simulation along the phase diagram scan

N	N_W	x_{mol}	x_{mass}	n	N_W	x_{mol}	x_{mass}	n	N_W	x_{mol}	x_{mass}
1	112890	0.0070	0.0903	58	10917	0.0683	0.5064	115	963	0.4538	0.9208
2	108369	0.0073	0.0937	59	10476	0.0709	0.5167	116	921	0.4648	0.9240
3	104031	0.0076	0.0972	60	10053	0.0737	0.5270	117	879	0.4765	0.9272
4	99864	0.0079	0.1008	61	9645	0.0766	0.5373	118	840	0.4878	0.9302
5	95865	0.0083	0.1046	62	9255	0.0796	0.5475	119	801	0.4997	0.9333
6	92025	0.0086	0.1085	63	8880	0.0826	0.5578	120	765	0.5112	0.9361
7	88338	0.0090	0.1125	64	8520	0.0858	0.5680	121	729	0.5232	0.9389
8	84801	0.0093	0.1167	65	8175	0.0891	0.5781	122	696	0.5348	0.9415
9	81405	0.0097	0.1209	66	7842	0.0926	0.5882	123	663	0.5468	0.9441
10	78144	0.0101	0.1254	67	7524	0.0961	0.5982	124	633	0.5583	0.9465
11	75015	0.0106	0.1299	68	7218	0.0998	0.6081	125	603	0.5702	0.9489
12	72009	0.0110	0.1346	69	6924	0.1036	0.6180	126	573	0.5827	0.9513
13	69123	0.0114	0.1394	70	6642	0.1075	0.6277	127	546	0.5944	0.9535
14	66354	0.0119	0.1444	71	6372	0.1115	0.6374	128	519	0.6065	0.9557
15	63696	0.0124	0.1495	72	6114	0.1157	0.6469	129	495	0.6178	0.9577
16	61143	0.0129	0.1548	73	5865	0.1200	0.6563	130	471	0.6294	0.9596
17	58692	0.0134	0.1602	74	5625	0.1245	0.6657	131	447	0.6415	0.9616
18	56340	0.0140	0.1658	75	5394	0.1292	0.6749	132	426	0.6525	0.9634
19	54081	0.0146	0.1716	76	5175	0.1339	0.6840	133	405	0.6639	0.9651
20	51912	0.0152	0.1775	77	4962	0.1388	0.6930	134	384	0.6757	0.9669
21	49830	0.0158	0.1835	78	4758	0.1439	0.7018	135	363	0.6879	0.9686
22	47832	0.0165	0.1897	79	4563	0.1492	0.7105	136	345	0.6987	0.9701
23	45915	0.0171	0.1961	80	4377	0.1545	0.7190	137	327	0.7098	0.9716
24	44073	0.0178	0.2026	81	4197	0.1601	0.7274	138	309	0.7214	0.9732
25	42306	0.0186	0.2093	82	4026	0.1658	0.7356	139	291	0.7333	0.9747
26	40608	0.0193	0.2162	83	3861	0.1716	0.7436	140	276	0.7435	0.9759
27	38979	0.0201	0.2232	84	3702	0.1777	0.7516	141	261	0.7540	0.9772
28	37416	0.0209	0.2304	85	3549	0.1840	0.7594	142	246	0.7648	0.9785
29	35916	0.0218	0.2377	86	3402	0.1904	0.7670	143	231	0.7759	0.9798
30	34476	0.0227	0.2452	87	3261	0.1970	0.7745	144	216	0.7874	0.9811
31	33093	0.0236	0.2529	88	3126	0.2038	0.7818	145	204	0.7968	0.9821
32	31764	0.0246	0.2607	89	2997	0.2107	0.7889	146	192	0.8065	0.9831
33	30489	0.0256	0.2687	90	2874	0.2177	0.7958	147	180	0.8163	0.9842
34	29265	0.0266	0.2768	91	2754	0.2251	0.8026	148	168	0.8264	0.9852
35	28089	0.0277	0.2851	92	2640	0.2326	0.8092	149	156	0.8368	0.9863
36	26961	0.0288	0.2935	93	2529	0.2403	0.8158	150	144	0.8475	0.9873
37	25878	0.0300	0.3021	94	2424	0.2481	0.8221	151	135	0.8556	0.9881
38	24837	0.0312	0.3108	95	2322	0.2562	0.8283	152	126	0.8639	0.9889
39	23838	0.0325	0.3197	96	2226	0.2644	0.8342	153	117	0.8724	0.9897
40	22881	0.0338	0.3286	97	2133	0.2728	0.8400	154	108	0.8811	0.9904
41	21960	0.0351	0.3378	98	2043	0.2814	0.8457	155	99	0.8899	0.9912
42	21078	0.0366	0.3470	99	1956	0.2903	0.8513	156	90	0.8989	0.9920
43	20229	0.0380	0.3564	100	1872	0.2994	0.8568	157	81	0.9081	0.9928
44	19416	0.0396	0.3658	101	1794	0.3084	0.8619	158	72	0.9174	0.9936
45	18636	0.0412	0.3754	102	1719	0.3176	0.8669	159	66	0.9238	0.9941

46	17886	0.0428	0.3851	103	1647	0.3269	0.8718	160	60	0.9302	0.9947
47	17166	0.0445	0.3948	104	1578	0.3364	0.8765	161	54	0.9368	0.9952
48	16476	0.0463	0.4047	105	1509	0.3465	0.8813	162	48	0.9434	0.9957
49	15813	0.0482	0.4146	106	1443	0.3567	0.8859	163	42	0.9501	0.9963
50	15177	0.0501	0.4246	107	1380	0.3670	0.8903	164	36	0.9569	0.9968
51	14565	0.0521	0.4347	108	1320	0.3774	0.8946	165	30	0.9639	0.9973
52	13977	0.0541	0.4449	109	1263	0.3878	0.8987	166	24	0.9709	0.9979
53	13413	0.0563	0.4550	110	1209	0.3982	0.9026	167	18	0.9780	0.9984
54	12873	0.0585	0.4653	111	1155	0.4092	0.9065	168	12	0.9852	0.9989
55	12354	0.0608	0.4755	112	1104	0.4202	0.9103	169	6	0.9926	0.9995
56	11856	0.0632	0.4858	113	1056	0.4310	0.9138	170	0	1.0000	1.0000
57	11376	0.0657	0.4961	114	1008	0.4425	0.9174				

* NW gives the total amount of water particles, being the amount of each type equal to $N_W/3$. The molar fraction reported is the equivalent in a real system, where the number of real water molecules corresponds to 3 times N_W.
